# Regulation and Novel Action of Thymidine Phosphorylase in Non-Small Cell Lung Cancer: Crosstalk with Nrf2 and HO-1

**DOI:** 10.1371/journal.pone.0097070

**Published:** 2014-05-12

**Authors:** Magdalena Tertil, Klaudia Skrzypek, Urszula Florczyk, Kazimierz Weglarczyk, Halina Was, Guillaume Collet, Alan Guichard, Tomasz Gil, Jaroslaw Kuzdzal, Alicja Jozkowicz, Claudine Kieda, Chantal Pichon, Jozef Dulak

**Affiliations:** 1 Department of Medical Biotechnology, Faculty of Biochemistry, Biophysics and Biotechnology, Jagiellonian University, Krakow, Poland; 2 Centre de Biophysique Moleculaire, CNRS UPR4301, Orléans, France; 3 Department of Molecular Neuropharmacology, Institute of Pharmacology, Polish Academy of Sciences, Krakow, Poland; 4 Department of Thoracic Surgery, Jagiellonian University Medical College, John Paul II Hospital, Krakow, Poland; Children's Hospital Boston & Harvard Medical School, United States of America

## Abstract

Proangiogenic enzyme thymidine phosphorylase (TP) is a promising target for anticancer therapy, yet its action in non-small cell lung carcinoma (NSCLC) is not fully understood. To elucidate its role in NSCLC tumor growth, NCI-H292 lung mucoepidermoid carcinoma cells and endothelial cells were engineered to overexpress TP by viral vector transduction. NSCLC cells with altered expression of transcription factor Nrf2 or its target gene heme oxygenase-1 (HO-1) were used to study the regulation of TP and the findings from pre-clinical models were related to gene expression data from clinical NSCLC specimens. Overexpression of Nrf2 or HO-1 resulted in upregulation of TP in NCI-H292 cells, an effect mimicked by treatment with an antioxidant N-acetylcysteine and partially reversed by HO-1 knockdown. Overexpression of TP attenuated cell proliferation and migration *in vitro*, but simultaneously enhanced angiogenic potential of cancer cells supplemented with thymidine. The latter was also observed for SK-MES-1 squamous cell carcinoma and NCI-H460 large cell carcinoma cells. TP-overexpressing NCI-H292 tumors *in vivo* exhibited better oxygenation and higher expression of IL-8, IL-1β and IL-6. TP overexpression in endothelial cells augmented their angiogenic properties which was associated with enhanced generation of HO-1 and VEGF. Correlation of TP with the expression of HO-1 and inflammatory cytokines was confirmed in clinical samples of NSCLC. Altogether, the increased expression of IL-1β and IL-6 together with proangiogenic effects of TP-expressing NSCLC on endothelium can contribute to tumor growth, implying TP as a target for antiangiogenesis in NSCLC.

## Introduction

Lung tumors rank as the top cause of cancer-related deaths worldwide, with non-small cell lung cancer (NSCLC) being the most prevalent. NSCLC patients are often diagnosed with advanced disease, when systemic chemotherapy is the major therapeutic option. Since tumor growth and metastasis are dependent on angiogenesis, mechanisms governing new blood vessel formation have been targeted for intervention in lung cancer [1]. However, addition of anti-VEGF agents to conventional chemotherapy resulted only in slight improvement of median survival [1], [2] with patients experiencing tumor recurrence due to emergence of drug resistance to antiangiogenic agents, underlining an urgent need for new targets for combinatorial treatments.

Thymidine phosphorylase (TP, E.C.2.4.2.4) is a pyrimidine salvage synthesis pathway enzyme, which is also known for its proangiogenic properties. TP catalyzes reversible phosphorolysis of thymidine into thymine and 2-deoxy-D-ribose-1-phosphate (dRP), which is further dephosphorylated to 2-deoxy-D-ribose (dR). The enzyme and its sugar products stimulate endothelial cell migration and tube formation *in vitro* and enhance angiogenesis in various models *in vivo* [3]. TP is frequently overexpressed in human tumors, including NSCLC [3], [4] and has been shown to correlate with higher microvessel density, more advanced tumor stage, metastasis and poor prognosis [3]. Proangiogenic action of TP in tumors, apart from the direct action of its products on endothelial cells, may also involve the stimulation of expression of other angiogenic factors such as VEGF, interleukin-8 (IL-8) or heme oxygenase-1 (HO-1) [5], [6]. Consequently, targeting TP with small-molecule inhibitors is currently investigated as a novel antiangiogenic strategy [7]. Nevertheless, for the development of effective combinatorial chemotherapeutics, retaining enzymatic activity of TP may be necessary as it catalyzes an important step in activation of fluoropyrimidine-based agents such as capecitabine, which has been proposed as alternative treatment for advanced NSCLC [8]. This dual function of TP in tumor growth and therapy implies that inhibiting protumoral effects of the enzyme may require targeting its downstream mediators. Elucidating the mechanisms of regulation and tumor-promoting actions of TP is therefore of crucial importance.

Nrf2 (nuclear factor (erythroid-derived 2)-like 2) is a transcription factor regulating cellular antioxidant responses [9]. It is frequently constitutively active in tumors, including lung cancer, and may be further induced by anticancer treatments. It drives expression of cytoprotective genes leading to the development of resistance to cytotoxic agents [10]. One of Nrf2 targets is HO-1, which converts heme into CO, ferrous iron and biliverdin, and which has been demonstrated to mediate Nrf2-driven resistance of NSCLC cells to chemotherapy [11], [12]. Interestingly, both proteins play roles in promotion of angiogenesis: the action of HO-1 upstream and downstream angiogenic VEGF and SDF1α is well established [13], and the involvement of Nrf2 in regulation of angiogenic IL-8 has been demonstrated [14]–[16].

Here we investigate the biological role of TP focusing on angiogenesis and the interplay with Nrf2 and HO-1 in non-small cell lung cancer and endothelial cells. Our results show the effects of TP overexpression in NSCLC cells *in vitro* and *in vivo* and highlight the importance of proangiogenic action of the enzyme.

## Materials and Methods

### Plasmids and viral vectors

Plasmid pBK-RSV-TP harboring human TP cDNA was kindly provided by Dr. S. Liekens (Rega Institute for Medical Research, K.U. Leuven, Belgium). Plasmid pEF(Blue)-Nrf2 containing human Nrf2 cDNA was kindly gifted by Dr. J.A. Johnson (Division of Pharmaceutical Sciences, University of Wisconsin-Madison, USA) [17]. Construction of retroviral vectors (RVs) RV-TP and RV-Nrf2 was conducted as described in Supplementary Methods (File S1). Retroviral plasmid pMSCV-Luc containing luciferase expression cassette for production of RV-Luc was obtained from Addgene. All RVs including a control RV-empty vector (LNCX2) were produced as described in [18].

Adenoviral vectors (AdVs) harboring TP cDNA (AdTP) were developed as described in Supplementary Methods (File S1) and control vectors with GFP (AdGFP) as reported previously [16].

### Cell lines and culture conditions

Human NSCLC cell lines: NCI-H292 (mucoepidermoid carcinoma, purchased from ATCC), A549 (adenocarcinoma, obtained from Prof. Jakub Golab, Warsaw Medical University, Warsaw, Poland) and NCI-H460 (large cell carcinoma, purchased from ATCC) were cultured in RPMI 1640 (PAA) and SK-MES-1 (squamous cell carcinoma, purchased from ATCC) was cultured in MEM (Gibco), each supplemented with 10% fetal bovine serum (PAA) and penicillin (100 U/mL)/streptomycin (10 µg/mL) (Sigma) (pen/strep). Human microvascular endothelial cells (HMEC-1, obtained from Dr Francis Candal, Center for Disease Control and Prevention, Atlanta, USA) were cultured in MCDB 131 supplemented with 10% FBS, L-glutamine 2 mM, pen/strep, EGF 10 ng/mL and hydrocortisone 1 mg/mL. Primary human umbilical vein endothelial cells (HUVEC) were isolated as described previously [19] and cultured in M199 (PAA) supplemented with 20% FBS, pen/strep and endothelial cell growth supplement ECGS 30 mg/L (Millipore).

All cells were maintained in standard culture conditions: 37°C, 5% CO_2_, 95% humidity. For the investigation of the effects of hypoxia cells were placed for 24 or 48 hours in a chamber (Biospherix USA) under controlled gas atmosphere 1% O_2_, 5% CO_2_ and 94% N_2_ placed at 37°C in a cell culture incubator.

For establishment of cell lines NCI-H292-Luc-TP (NCI-TP) and NCI-H292-Luc-Nrf2 (NCI-Nrf2) stably overexpressing luciferase and respective transgenes and control NCI-H292-Luc-EV (NCI-EV) modified with empty vector, cells were transduced with retroviral vectors. First, an infection with RV-transgene or RV-empty vector (RV-EV) was performed and stably transformed cells were selected by geneticin (1 mg/mL), which was followed by transduction with RV-Luc and selection by hygromycin (0.3 mg/mL). NCI-H292-Luc-HO-1 (NCI-HO1) cell line was developed and validated earlier in our laboratory [20]. For maintenance of transgene expression cells were routinely kept in standard medium additionally supplemented with geneticin (0.5 mg/mL) and hygromycin (0.1 mg/mL). For experiments cells were seeded in medium without antibiotics.

### Transient TP overexpression and stimulation of cells with NAC or TP substrate

Thymidine (Thd) and N-acetylcysteine (NAC) were purchased from Sigma Aldrich. For transient TP overexpression in ECs, HMEC-1 and HUVEC cells were transduced with adenoviral vectors AdTP or control AdGFP at MOI = 10 for 24 h and then stimulated with Thd for additional 24 h in complete medium. For transient TP overexpression in NSCLC cells, SK-MES-1 and NCI-H460 were transduced at MOI = 20 and MOI = 40, respectively, and stimulated with 1 mM Thd in medium supplemented with 2% FBS 48h post-transduction.

### Real-time RT PCR

mRNA levels of genes were determined by quantitative RT PCR. RNA was isolated with either Qiazol (Qiagen) or using RNeasy Plus Micro Kit (Qiagen) according to the manufacturer's instructions. 1 µg of RNA was reverse-transcribed into cDNA using oligo-dT primers with RevertAid Premium First Strand cDNA Synthesis Kit (Fermentas). Real-time PCR was performed using 30 ng of sample with QuantiTect SYBR Green (Qiagen, analysis of *in vitro* experiments) or SYBR Premix Ex Taq II (Takara, analysis of *in vivo* experiments) according to the manufacturer's instructions on LightCycler 480 II system (Roche). Gene expression was calculated according to ΔCt or ΔΔCt methods with EF2 as a reference gene, with error bars calculated as standard deviations of the means, divided by √(N-1), where N was the number of independent experiments. Error propagation was not taken into account, what is some limitation of our study.

### Western blot analysis and ELISA

Western blot for HO-1 was performed as in [19]. For the detection of human TP mouse mAb P-GF.44C (Calbiochem) was used. Production of human VEGF and IL-8 in culture media and human IL-8, IL-1β and IL-6 in tumor lysates was quantified using DuoSet ELISA Kits (R&D) according to the manufacturer's protocols. Total protein concentration in the samples was measured by BCA method. Data were normalized by pre-assay dilutions of tumor lysates to an equal concentration of 1 mg/mL.

### Reporter gene assay for measurement of Nrf2 transcriptional activity

Assay was performed as already described in [15].

### Gene silencing experiment

Cells were transfected with 50 nM siRNA (Stealth RNAi HO-1 siRNA and control Stealth RNAi Negative Control siRNA, Invitrogen) using Lipofectamine 2000 reagent (Invitrogen) according to the manufacturer's instructions.

### Cell proliferation and migration

Cell proliferation was measured by colorimetric assay of BrdU incorporation (Cell Proliferation ELISA, Roche) according to the vendor's instruction. In order to investigate cell migration, cells were grown to full confluence and serum-starved for 24 h to block proliferation. Scratch was made on the monolayer with a pipette tip. Migration was followed by time lapse microscopy (Zeiss Axiovert 200 M) for 6-8 different fields for each condition. Mean covered area was calculated at different time points and expressed as the percentage of scratch area at time zero using ImageJ software.

### Angiogenesis assays *in vitro*

Tube formation assay on Matrigel was performed as already described [21]. NSCLC cells were incubated in medium containing 2% FBS in normoxia and hypoxia for 48 h, conditioned medium was collected and mixed at 1∶1 vol/vol with MCDB 131 supplemented with 2% FBS and antibiotics without other additives for stimulation of HMEC-1 or HUVEC. Spheroid assay on HUVEC cells was performed as previously [19].

### Animal experiments

#### Ethics statement

All animals were handled in strict accordance with good animal practice and all animal work was approved by the CNREEA 03 (Comité national de réflexion éthique sur l'expérimentation animale) Campus CNRS d'Orléans Ethics Committee in France.

6-week old female athymic Swiss nude mice were purchased from Charles River (France). For establishment of NCI-TP and control NCI-EV tumor xenografts, exponentially growing cells were harvested using Cell Dissociation Solution (Sigma) and resuspended in PBS. 5×10^6^ cells in 100 µL were injected subcutaneously into the right leg of each mouse (10 animals/cell line). Tumor growth was monitored for 5 weeks by caliper measurements, tumor volume was calculated according to the formula *V* = *D*×*d* ^2^×0.5 (*V* is the tumor volume, *D* is the biggest dimension; *d* is the smallest dimension).

### Measurement of tumor oxygenation

Oxygenation of tumor tissue was measured by OxyLite sensor system based on ruthenium fluorescence quenching by O_2_ (Oxford Optronix).

### Analysis of clinical material

#### Ethics statement

Human studies were approved by Local Ethical Committee of the Collegium Medicum of the Jagiellonian University in Krakow, Poland. Patients have expressed their written consent to participation in the study.

Biopsies of primary tumors and tumor metastases to lymph nodes (if present) were collected during surgery from 24 patients suffering from NSCLC adenocarcinoma. Patients were treated at the Clinic of Thoracic Surgery, Jagiellonian University Medical College, 31-202 Kraków, Poland.

### Statistical analysis

Unless stated otherwise, results show mean ± SEM of at least 3 independent experiments performed in duplicates. Unpaired Student's t-tests were used to assess whether the means of two groups differed significantly. For comparison of multiple groups one-way ANOVA analysis with Tukey post-test was employed. Differences with a value of p<0.05 were considered statistically significant.

## Results

### TP is differentially expressed in NSCLC cells with varying levels of Nrf2 and heme oxygenase-1

**Figure 1:**
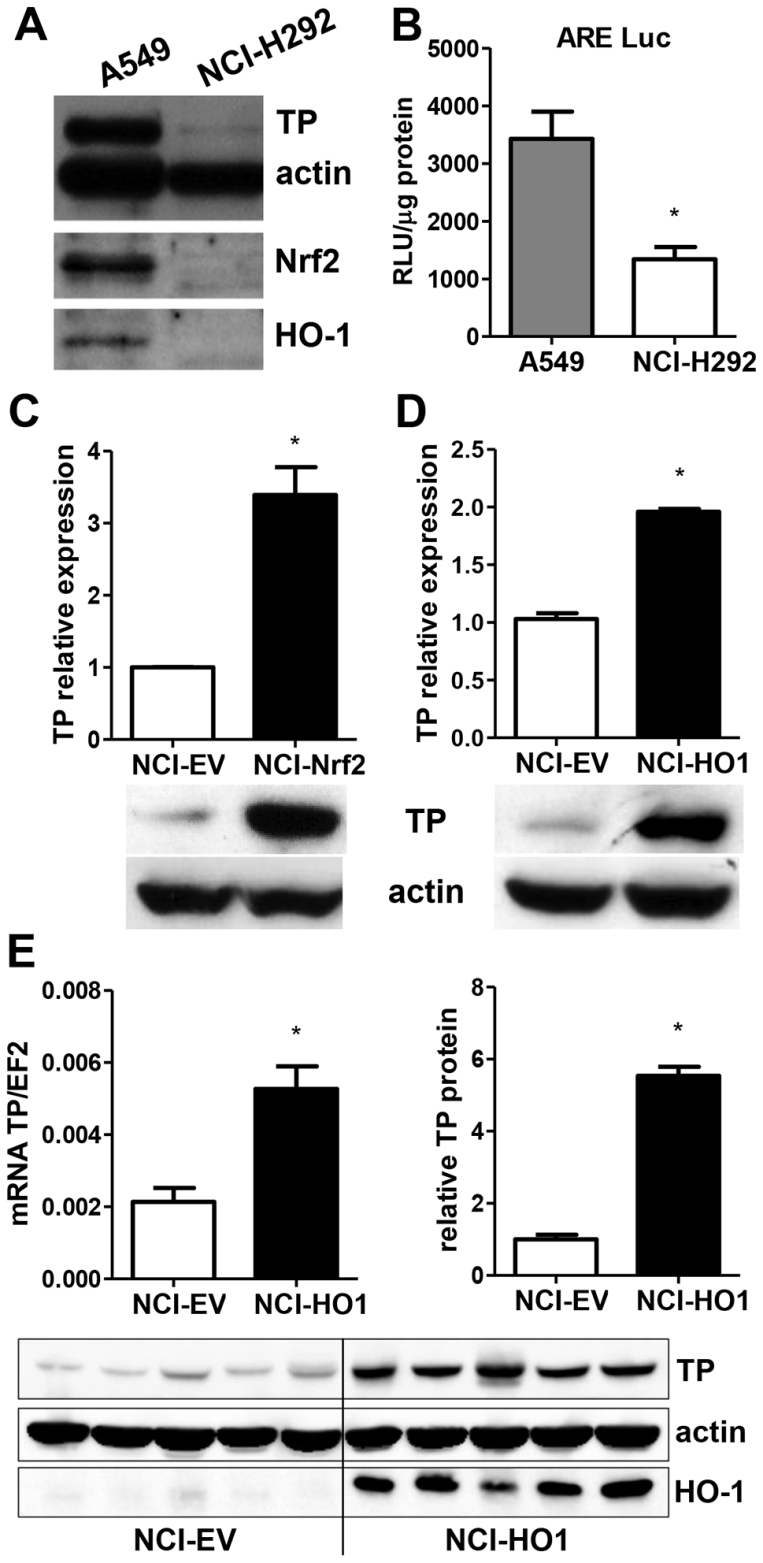
Effect of differential Nrf2/HO-1 expression on TP expression in NSCLC *in vitro* and *in vivo*. **A**. Basal TP, Nrf2 and HO-1 protein levels in A549 and NCI-H292 NSCLC cell lines indicating possible association of TP expression with Nrf2/HO-1 axis. **B**. Basal Nrf2 transcriptional activity in NSCLC cell lines measured by reporter gene assay using plasmid harboring luciferase gene under the control of antioxidant response element (ARE) (n = 3, *p<0.05 NCI-H292 *vs* A549). **C–D**. Increased TP mRNA and protein expression in NCI-H292 cell lines stably overexpressing Nrf2 or HO-1 (n = 4, *p<0.05 control empty vector (EV) *vs* transgene overexpression) **E**. TP mRNA (left) and protein expression (right, densitometric analysis of WB in lower panel) *in vivo* in control and HO-1-overexpressing NCI-H292 xenografts (established as described in [20]) corroborates the *in vitro* data (n = 5, *p<0.05 NCI-HO1 *vs* NCI-EV).

We investigated TP expression in NSCLC cell lines A549 and NCI-H292 originating from different histological types of tumors – adenocarcinoma and mucoepidermoid carcinoma, respectively. Western blot analysis revealed that A549 adenocarcinoma displays high basal expression of TP, while the enzyme level is very low in NCI-H292 (Fig. 1A). As the two cell lines are known to differ in activity of transcription factor Nrf2 and its target gene HO-1, both being expressed at higher level in A549 than in NCI-H292 ([22], Fig. 1A,B), we investigated whether the Nrf2/HO-1 axis could be involved in the regulation of TP. Indeed, when Nrf2 and HO-1 were independently stably overexpressed in NCI-H292 cells (Fig. S1A, [20]), which have low basal Nrf2 and HO-1, the induction of TP was observed in both NCI-Nrf2 and NCI-HO1 cells (Fig. 1C&D, respectively). This expression pattern was also confirmed *in vivo* in subcutaneous HO-1-overexpressing xenograft tumors derived from NCI-HO1 cells in nude mice (Fig. 1E).

**Figure 2:**
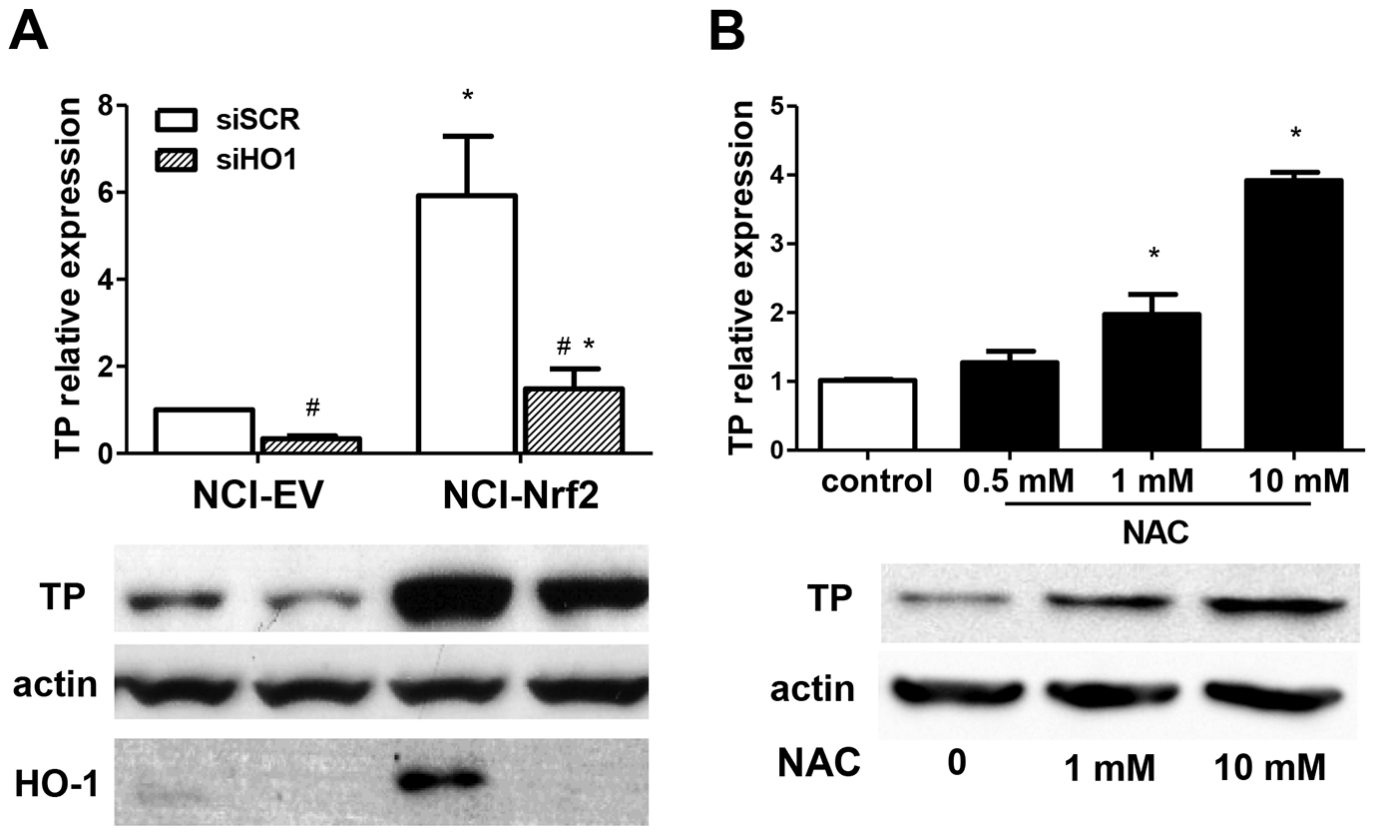
Regulation of TP by Nrf2/HO-1 axis in NCI-H292 cells. **A**. HO-1 and TP mRNA and protein expression in NCI-H292 cells with HO-1 knockdown. NCI-Nrf2 and NCI-EV control cells were transfected with 50 nM siRNA against HO-1 (siHO1) or control scrambled sequence (siSCR) for 72 h leading to downregulation of TP expression following HO-1 silencing. (n = 4, *p<0.05 NCI-Nrf2 *vs* NCI-EV, #p<0.05 siHO1 *vs* siSCR). **B**. Effect of antioxidant N-acetylcysteine (NAC) on TP expression. NCI-EV cells were stimulated with indicated concentrations of NAC for 24 h resulting in a dose-dependent upregulation of TP. (n = 3, * p<0.05 control *vs* stimulation).

Nrf2-binding sites within the TP promoter have not been identified (analysis not shown) suggesting that the regulation is indirect. Since HO-1 is a known target of Nrf2 and was significantly upregulated in NCI-Nrf2 cells (Fig. S1B), we next aimed to determine whether the effect of Nrf2 was HO-1-dependent. NCI-Nrf2 cells were transfected with siRNA against HO-1 (Fig. S2), which led to a partial downregulation of TP expression in both Nrf2-overexpressing cells and control cells transduced with empty vector (NCI-EV) (Fig. 2A), implying that HO-1 plays a role in the regulation of TP in NSCLC, yet its involvement in the effect of Nrf2 is minor. Moreover, treatment of NCI-EV cells with an antioxidant N-acetylcysteine resulted in a dose-dependent upregulation of TP (Fig. 2B), mimicking the regulation of the enzyme by Nrf2/HO-1 overexpression. Since the stimulation of control cells with HO-1 products failed to reproduce the upregulation of TP found in HO-1 overexpressing cells (Fig. S3), the effect of Nrf2/HO-1 could be attributed to attenuation of oxidative stress, as we have already shown that NCI-HO1 cells have lower level of reactive oxygen species [20].

### TP overexpression attenuates proliferation and migration of NCI-H292 cells but enhances angiogenic potential of NSCLC cell lines *in vitro*

**Figure 3:**
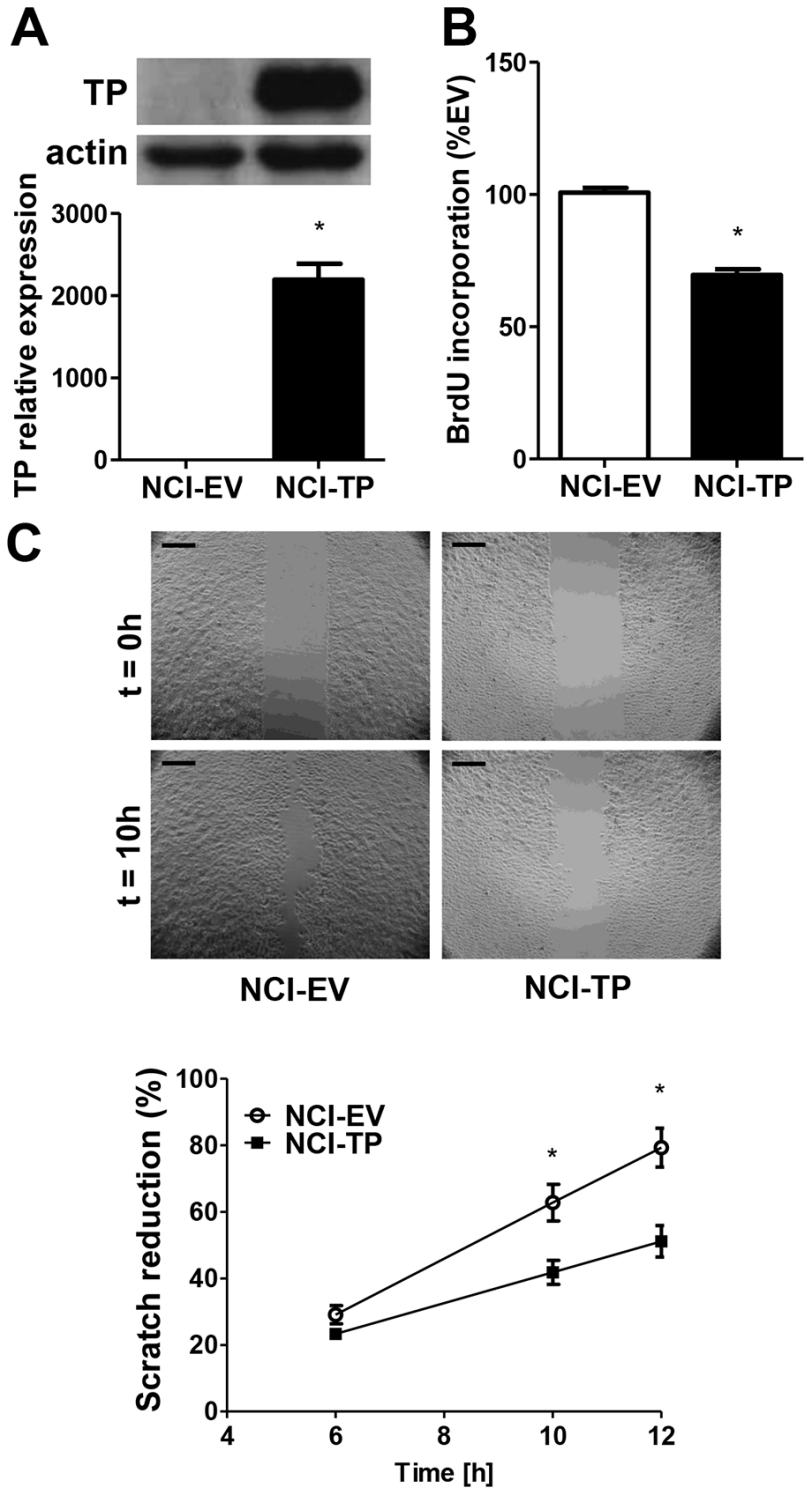
Effect of TP overexpression on proliferation and migration of NCI-H292 cells *in vitro*. **A**. Validation of the model – upregulation of TP mRNA and protein levels in NCI-H292 cell line stably overexpressing TP, established as described in Materials and Methods (n = 3). **B**. Relative basal proliferation rates of NCI-EV and TP-overexpressing cells measured by incorporation of bromodeoxyuridine (BrdU) (n = 4). **C**. Basal migration rates of NCI-EV and NCI-TP cells determined by scratch assay (n = 4) (scale bar – 200 µm) * p<0.05 NCI-TP vs NCI-EV

Next, we aimed to investigate the direct effects of TP itself on proliferation and migration of NCI-H292 cells. A stable cell line overexpressing both luciferase and TP (NCI-TP) was established by retroviral transduction and validated for TP expression (Fig. 3A). Unexpectedly, proliferation of NCI-TP cells was inhibited (Fig.3B). Scratch assay showed that migratory potential of the NCI-TP cells was also attenuated (Fig. 3C, File S2 & S3). Downregulation of mRNA levels of matrix metalloproteinases (MMPs) MMP-1 and MMP-2 was also observed (Fig. S4) that could potentially negatively affect tumorigenic potential of NCI-H292 cells *in vivo*.

**Figure 4:**
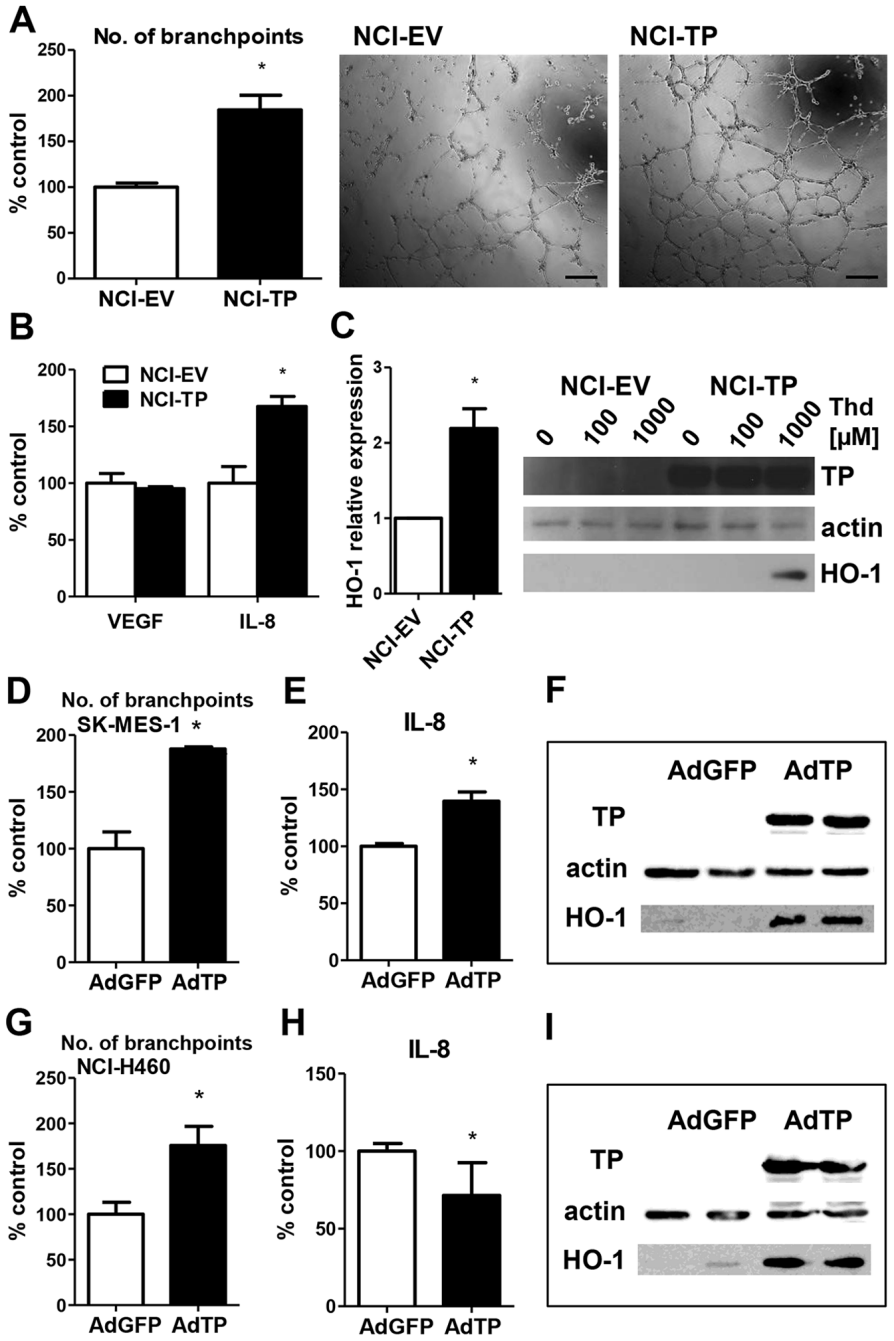
Effect of TP overexpression on angiogenic potential of NSCLC cells *in vitro*. **A**. Increased angiogenic potential of TP overexpressing cells in hypoxia in presence of thymidine. NCI-H292 cells were stimulated with 1 mM Thd for 48 h under 1% oxygen and conditioned media were applied on HMEC-1 cells seeded on Matrigel. The number of branchpoints formed by HMEC-1 treated with conditioned media from either empty-vector transduced NCI-H292 cells (NCI-EV) or TP-transduced (NCI-TP) has been calculated (scale bar – 200 µm) (n = 3). **B**. Production of angiogenic factors in TP-overexpressing NCI-H292 cells under hypoxia in presence of 1 mM Thd for 24h quantified by ELISA detecting upregulation of IL-8 in NCI-TP cell line (n = 4). **C**. Increased HO-1 mRNA (left) and protein (right) expression in TP-overexpressing cells in presence of 1 mM Thd in hypoxia after 24 h (n = 3). * p<0.05 NCI-TP vs NCI-EV. **D–I**. Enhanced angiogenic potential of lung squamous cell carcinoma SK-MES-1 and large cell carcinoma cells NCI-H460 following transient TP overexpression. SK-MES-1 (**D–F**, n = 3) and NCI-H460 (**G–I**, n = 4) cells were transduced with AdTP or control AdGFP for 48 h, stimulated with 1 mM Thd for additional 48 h under 1% oxygen (**D–E&G–H**) or normoxia (**F&I**). Conditioned media were used in Matrigel assay on HUVEC cells (**D&G**) and measurement of IL-8 production (**E&H**) and HO-1 expression was assayed in cell lysates (**F&I**). * p<0.05 AdTP vs AdGFP

Since the major role of TP in tumor growth is thought to be associated rather with its proangiogenic properties [23], we next focused on elucidating the role of TP in modulation of angiogenesis in our NCSLC model. Under standard conditions no effect of TP overexpression/TP products on angiogenic potential of tumor cells could be observed (Fig. S5). Nevertheless, the major trigger of angiogenic switch *in vivo* is oxygen deprivation. Therefore, to better mimic the environment of a growing tumor, we placed the cells under hypoxia and provided them with thymidine, which can be released from the necrotic core of tumor. While the inhibitory effects of TP overexpression on proliferation and migration in NSCLC cells were unaffected by either hypoxia and/or thymidine (Fig. S6), altered conditions revealed enhanced angiogenic potential of NCI-TP cells, as evidenced by Matrigel tube formation assay with conditioned media (Fig. 4A). It was associated with the upregulation of IL-8 protein production by tumor cells (Fig. 4B) and the induction of HO-1 (Fig. 4C) that could be indicative of elevated oxidative stress in TP-overexpressing cells, which is consistent with the findings from other tumor types [5]. Importantly, TP-overexpressing cells exhibited enhanced angiogenic potential in the presence of thymidine also under normoxic conditions (Fig. S7).

We next investigated whether TP modulates angiogenic potential of tumor cells originating from other NSCLC histological types, namely squamous cell carcinoma SK-MES-1 cell line and NCI-H460 large cell carcinoma. Conditioned media collected from hypoxic cells transiently overexpressing TP following adenoviral vector transduction and stimulation with Thd caused enhanced branching of endothelial cells in comparison to control AdGFP-transduced cells for both cell types (Fig. 4D,G), which was accompanied by increased production of IL-8 by SK-MES-1 cells (Fig. 4E). Induction of HO-1 by TP was observed under normoxic conditions (Fig. 4F,I).

### TP modulates expression of inflammatory cytokines *in vivo*

**Figure 5:**
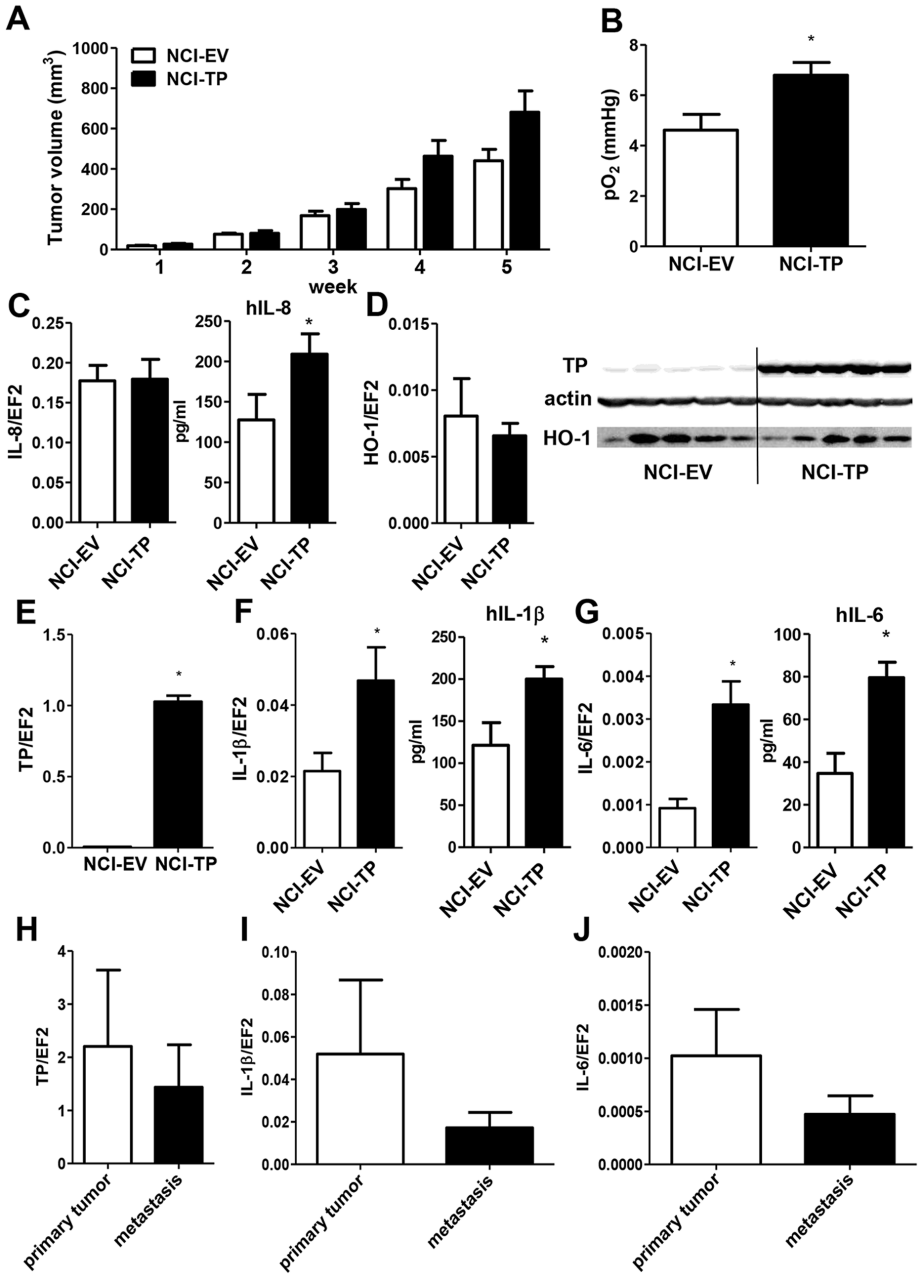
Effect of TP overexpression on NCI-H292 xenograft tumor growth and data from clinical NSCLC samples. **A**. Measurement of tumor volume by caliper. NCI-EV and NCI-TP cells were xenografted into nude mice as described in Materials and Methods. **B**. Measurement of tumor oxygenation at 5 weeks of tumor growth (n = 7 for NCI-EV, n = 9 for NCI-TP). **C–G**. mRNA (left) and protein (right) expression of human IL-8 (**C**), HO-1 (**D**), TP (**D,E**) and inflammatory cytokines (**F–G**) in xenograft tumors indicates upregulation of secretion of IL-8, IL-1β and IL-6 in NCI-TP xenografts (n = 5, *p<0.05 NCI-TP vs NCI-EV). **H–J**. mRNA expression of TP (**H**) and inflammatory cytokines (**I–J**) in biopsies from human primary and secondary lung adenocarcinomas.

To determine how the complex effects of TP overexpression on proliferation, migration and angiogenic potential of NCI-H292 cells observed *in vitro* would affect tumor development *in vivo*, tumor cells were implanted subcutaneously into nude mice and tumor growth was monitored for 5 weeks. TP overexpression tended to accelerate growth of the NSCLC xenografts (Fig. 5A) (p<0.1 NCI-TP *vs* NCI-EV at 4 and 5 weeks). Moreover, NCI-TP tumors showed an insignificant trend (p = 0.2) towards enhanced invasion of tumor-draining lymph nodes (Fig. S8). As cell proliferation and migration are impaired by TP overexpression (Fig. 3B-C), we presumed the differences in angiogenic properties. Indeed, measurement of tumor oxygenation performed at the end of the experiment showed that NCI-TP tumors were significantly better oxygenated than tumors derived from control cells, which coincided with increased production of hIL-8 in the former tumors (Fig. 5B,C). Importantly, in our previous research using the NCI-H292 xenografts we have shown that enhanced tumor oxygenation corroborates enhanced tumor vascularization in this model [20]. Nevertheless, although the TP overexpression was confirmed to be retained in xenografts (Fig. 5D,E), no differences in levels of either HO-1 or other angiogenic factors could be detected in comparison with control tumors (Fig. 5D, Fig. S9A-D). To better understand the mechanism underlying the effect of TP overexpression on NSCLC *in vivo*, we determined the expression of inflammatory cytokines in the tumors. Human interleukin-1β and interleukin-6 levels were significantly increased in NCI-TP tumors (Fig. 5F&G). Also the expression of TNFα tended to be higher in TP-overexpressing tumors, but it did not reach statistical significance (Fig. S9E).

### Interplay of TP, HO-1 and inflammatory cytokines in clinical NSCLC specimens

We performed preliminary validation of our findings using clinical material of primary tumors and tumor-infiltrated lymph nodes collected during surgery from patients suffering from lung adenocarcinoma. While there were no differences in either TP, IL-1β or IL-6 expression between the primary and secondary tumor specimens (Fig.5 H-J), it is noteworthy that the basal expression of TP was high relative to constitutive gene EF2 in tumor tissue (mean TP/EF2 = 2.202), which is comparable with the TP/EF2 levels obtained in our TP-overexpressing xenografts (mean TP/EF2 = 1.027, see Fig. 5E). The relative gene/EF2 ratios are also similar for the interleukins, which shows that our xenograft model closely paralleled clinical situation. We found a significant correlation between HO-1 and TP expressions (Spearman Rank Correlation, R = 0.556; *p* = 0.028). What is more, a significant correlation of TP with IL-1β (R = 0.514, *p* = 0.001) and IL-6 (R = 0.519, *p* = 0.002) was observed thus confirming our data from the animal model showing a potential novel effect of TP upregulation in the carcinoma of the lung.

### TP overexpression enhances angiogenic potential of endothelial cells

**Figure 6:**
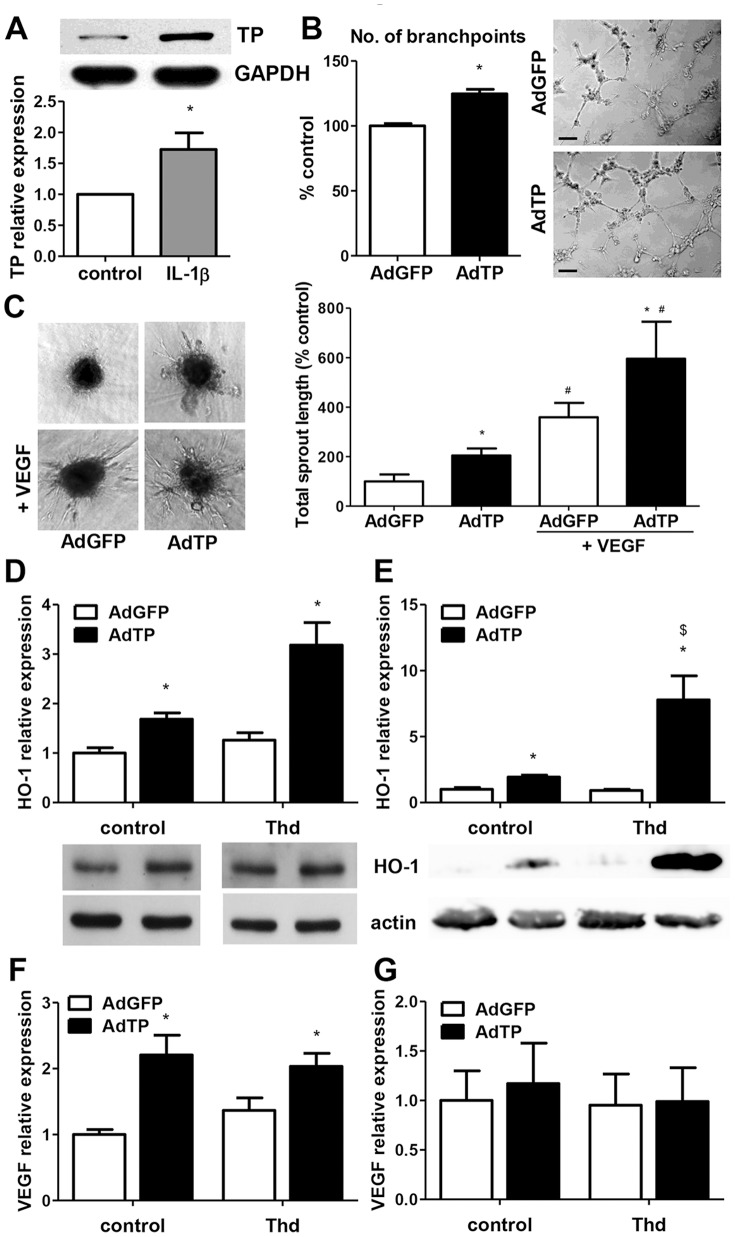
Effect of TP overexpression in endothelial cells on angiogenic potential. **A**. Effect of IL-1β on TP expression in HMEC-1 cells. Stimulation with 0.3 ng/mL human recombinant IL-1β for 24 h led to upregulation of TP in endothelial cells (ECs). (n = 3, * p<0.05 control *vs* IL-1β). **B–C**. ECs were transduced with adenoviral vectors AdTP or control AdGFP at MOI  =  10 for 48 h when Matrigel assay on HUVEC in the presence of VEGF (50 ng/mL) (**B**) and spheroid assay on HUVEC (VEGF 50 ng/mL) (**C**) was performed, indicating enhanced angiogenic potential of TP-overexpressing cells (n = 3, *p<0.05 AdTP *vs* AdGFP, # p<0.05 control *vs* VEGF) (scale bar – 100 µm) **D–G**. 24 h post-transduction with AdV endothelial cells were stimulated with 1 mM Thd for next 24 h. Analysis of HO-1 expression by quantitative PCR and western blot in HMEC-1 (**D**) and in HUVEC (**E**) shows induction of HO-1 in TP-overexpressing ECs, associated with increased VEGF expression in HMEC-1 (**F**) while there is no effect in HUVEC (**G**). (n = 4, * p<0.05 AdTP *vs* AdGFP, $ p<0.05 control *vs* Thd).

Interleukin-1β was demonstrated to induce expression of TP in primary macrovascular human endothelial cells HUVEC [24]. We reproduced this effect in a human microvascular endothelial cell line HMEC-1 (Fig. 6A), suggesting the regulation was common for different endothelial cell types. This observation raised a question whether TP-dependent upregulation of inflammatory cytokines in cancer cells could possibly contribute to modulation of tumor angiogenesis through modulation of TP in endothelium. Therefore, next we investigated the effects of TP overexpression in ECs. Introduction of TP into HUVEC enhanced the abilities of endothelial cells to form tubule-like structures in Matrigel and angiogenic sprouting in collagen gel (Fig. 6B,C). This was accompanied by induction of proangiogenic HO-1 (Fig. 6D,E), which was further potentiated in HUVEC cells in the presence of excess of TP substrate (Fig. 6E). In HMEC-1 model, a concomitant increase in VEGF production was observed following TP overexpression (Fig. 6F), while no effect could be observed for HUVEC (Fig. 6G), which may not release VEGF [25]–[27]. These results imply a novel proangiogenic action of TP within ECs, possibly through induction of other angiogenic proteins. The mechanism, however, may be strictly endothelial cell-type specific.

## Discussion

**Figure 7:**
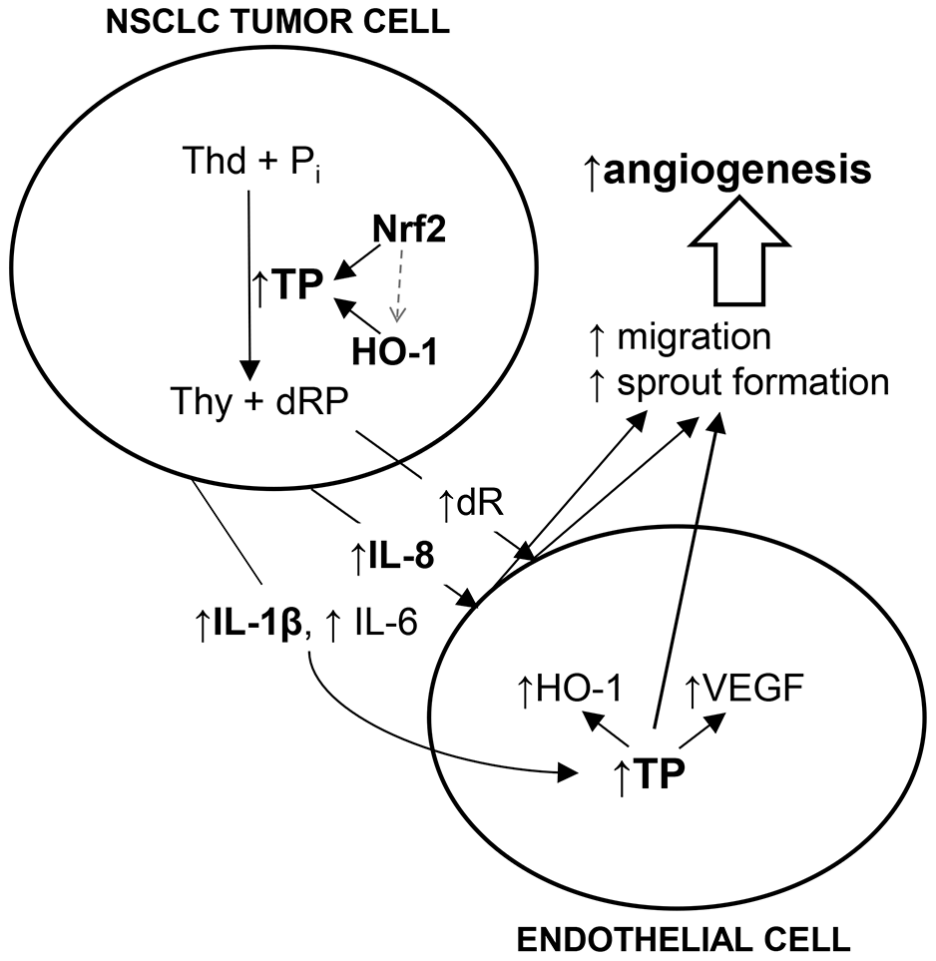
Putative regulation and proangiogenic action of thymidine phosphorylase in NSCLC NCI-H292 cells. (Thd – thymidine, Thy – thymine, dRP – 2-deoxyribose-1-phosphate, dR – 2-deoxyribose)

The salient finding of the present study is the demonstration that TP can be upregulated in NCI-H292 cells by activation of Nrf2/HO-1 pathway possibly through amelioration of oxidative stress. Induction of TP attenuated cell proliferation and migration *in vitro*. On the other hand, increased expression of IL-1β and IL-6 in TP-expressing tumors, as well as enhanced proangiogenic effects of TP-expressing NSCLC cells on endothelial cells, have been observed (Fig. 7).

In 2009, Lu *et al*. published the proof-of-concept *in vivo* study showing the feasibility of direct thymidine phosphorylase inhibition as a novel approach to antiangiogenesis in NSCLC [28]. Translation of these findings into clinic, however, requires extended knowledge concerning the biological roles of the enzyme. Here, we provide novel insights into the regulation and protumoral actions of TP in non-small cell lung cancer.

We have demonstrated the regulation of thymidine phosphorylase by Nrf2 and HO-1 in mucoepidermoid carcinoma of the lung, as the manipulation of Nrf2 or HO-1 levels led to concomitant modulation of TP levels *in vitro* and *in vivo* and HO-1 expression correlated with TP in clinical NSCLC specimens. To our knowledge, this is the first observation linking TP with Nrf2 and indicating that HO-1 may act upstream of TP in cancer. Thus far, coexpression of TP and HO-1 has already been reported in clinical specimens of human malignant vertical growth melanomas in macrophages [29]. Studies *in vitro* implicated that HO-1 could only play a role downstream of TP, as was described in vascular smooth muscle cells or bladder carcinoma, where TP overexpression resulted in induction of HO-1 expression [5], [30]. We provide here mechanistic data showing that the regulation is indirect, potentially involving modulation of oxidative status of the cell, as the effects of Nrf2 or HO-1 on TP expression were mimicked by treatment of control cells with an antioxidant N-acetylcysteine. Our data indicate that, apart from its established role in cancer biology, Nrf2 may also be an important player in regulation of tumor angiogenesis by enhancing the expression of proangiogenic TP, thus warranting further research into the Nrf2/HO-1-TP association in other tumor types. Moreover, positioning TP downstream of Nrf2 and HO-1 would have interesting implications for the design of new anticancer treatments. HO-1 itself is a validated therapeutic target as it is proangiogenic, antiapoptotic and generally cytoprotective enzyme which plays an important role in tumor development and therapy [31]. In NSCLC both HO-1 and TP, as well as Nrf2, are known to contribute to cell resistance to cisplatin, chemotherapeutic drug commonly used as a first-line treatment in clinic [12], [22], [32]. In light of our findings, targeting Nrf2 and/or HO-1 pathway would possibly concomitantly affect TP, thus broadening inhibitory action on another pathway.

Unexpectedly, we observed inhibition of the proliferation of NCI-H292 cells overexpressing TP *in vitro* and attenuation of their migratory potential. It was opposite to what has been observed in other tumor types including breast, cervical, bladder and gastric cancer (reviewed in [3]), showing that the effects of TP are cell type dependent. However, despite the inhibitory influence of TP on proliferation and migration of NSCLC cells *in vitro*, we show that *in vivo* the overexpression of the enzyme tended to accelerate tumor growth and increase local metastasis which was associated with significant upregulation of proinflammatory cytokines, interleukin-8 and better oxygenation of tumors. Accordingly, *in vitro* angiogenesis assays showed that in the presence of TP substrate, thymidine, NCI-TP cells enhanced the response of endothelial cells, an effect replicated also in two other NSCLC cell types: squamous carcinoma and large cell carcinoma. This broad proangiogenic effect could be exerted by at least two actions. First, it could be mediated directly by dRP and dR formed through TP pathway from Thd released from the necrotic core of tumor. It has been already shown that dR induces endothelial cell migration via activation of focal adhesion kinase and integrin signaling [33]. Second, TP may modulate expression of other angiogenic factors, as demonstrated in our study for interleukin-8 in NCI-H292 and SK-MES-1 cells, which has been previously associated with TP in other tumor types [5], [34], [35]. The upregulation of IL-8 *in vivo* could also be a mechanism of possible potentiation of TP expression within tumor microenvironment, as the chemokine was reported to induce TP in human cells [36]. However, we could not observe any difference in the expression of HO-1 or other angiogenic factors between TP-overexpressing and control tumors *in vivo*. It can be argued that additional modulation of angiogenic switch by TP could have occurred earlier in tumor development and was not possible to detect after 5 weeks of xenograft growth.

Nevertheless, the TP-overexpressing tumor cells exhibited enhanced expression of IL-1β and IL-6. To our knowledge, this is the first demonstration of such effect exerted by thymidine phosphorylase, which finds additional confirmation in the gene expression analysis in human clinical tumor specimens. Both IL-1β and IL-6-mediated inflammation may contribute to NSCLC-related morbidity and mortality [37], [38]. Moreover, the inflammatory cytokines can promote tumor growth and metastasis in an autocrine manner by enhancing cancer cell proliferation and activating pro-survival signaling pathways [39], [40]. Upregulation of IL-1β and IL-6 could potentially serve as autocrine positive feedback loop for TP in cancer cells and drive paracrine induction of the enzyme in tumor microenvironment. Stimulation of TP expression by these cytokines is a relatively well known pathway of its regulation and has been observed in many cell types (reviewed in [3]).

Indeed, here we confirmed that treatment with IL-1β results in upregulation of endogenous TP in endothelium. Since studies showing proangiogenic actions of TP on endothelial cells published so far only focused on the effects of exogenous recombinant TP and its sugar products, we overexpressed TP in HUVEC. Such intrinsic induction of TP in endothelium augmented their angiogenic properties, showing that paracrine induction of TP in endothelial cells by factors produced by TP-overexpressing cancer cells might also contribute to proangiogenic action of the enzyme in tumor microenvironment. Importantly, cells overexpressing TP displayed higher expression of another proangiogenic enzyme – HO-1. Our previous works [21], [41], as well as other studies [42], [43], have shown that HO-1 induces endothelial cell sprouting, plays an important role in mediating proangiogenic actions of VEGF and SDF-1 and induces VEGF expression [44]. Accordingly, in HMEC-1 cells VEGF expression paralleled HO-1 induction in response to upregulation of TP.

Taken together, our data show the regulation of TP expression by HO-1 and Nrf2 in NCI-H292 cells. Moreover, we demonstrate evidence for additional mechanism of protumoral action of TP involving upregulation of inflammatory cytokines and increased angiogenic response of endothelial cells, which provides further rationale for targeting this enzyme for antiangiogenesis in non-small cell lung carcinoma.

## Supporting Information

Figure S1 **Validation of transgene overexpression in NCI-Nrf2 stably transduced cell line.** **A**. Nrf2 mRNA in NCI-H292-Luc-Nrf2 (NCI-Nrf2) cell line developed as described in Materials and Methods. **B**. HO-1 mRNA in NCI-Nrf2 cell line. * p<0.05 NCI-Nrf2 vs NCI-EV.
(PDF)

Figure S2 **Validation of siRNA-mediated knockdown of HO-1 in NCI-H292 cells.** NCI-Nrf2 and NCI-EV control cells were transfected with 50 nM siRNA against HO-1 (siHO1) or control scrambled sequence (siSCR) for 72 h leading to downregulation of HO-1 mRNA expression. (n = 4, *p<0.05 NCI-Nrf2 *vs* NCI-EV, #p<0.05 siHO1 *vs* siSCR).
(PDF)

Figure S3 **Effect of HO-1 products on TP expression.** CORM-2 [CORM, CO-releasing molecule - tricarbonyldichlororuthenium(II) dimer], bilirubin and DMSO (used as solvent) were purchased from Sigma Aldrich. Inactive CORM (iCORM) was prepared by overnight evaporation of CORM stock solution. Biliverdin was from MP Bioscience, FeCl_3_ was from POCh. NCI-EV cells were stimulated with 10 µM CORM, iCORM, biliverdin, bilirubin or FeCl_3_ for 24 h (n = 3).
(PDF)

Figure S4 **Effect of TP overexpression on MMP expression.** qPCR analysis of MMP-1 and MMP-2 expression in control and TP-overexpressing NCI-H292 cells (n = 4). * p<0.05 NCI-TP vs NCI-EV.
(PDF)

Figure S5 **Effect of TP overexpression on angiogenic potential of NCI-H292 cells** ***in vitro*** **.** **A**. Basal angiogenic potential of TP overexpressing cells. Conditioned media (CM) were collected from unstimulated NCI-H292 cells under normoxia for 24 h. HUVEC spheroids were stimulated with CM or VEGF 10 ng/mL for 72 h (representative experiment, *p<0.05 control vs stimulation). **B**. Basal mRNA expression of angiogenic factors in TP-overexpressing cells in normoxia. **C**. mRNA expression of angiogenic factors in NCI-EV cells stimulated with TP products. Cells were stimulated with 200 µM 2-deoxyribose (dR)/2-deoxyribose-1-phosphate (dRP) for 24 h in normoxia.
(PDF)

Figure S6 **Effects of hypoxia and TP substrate on proliferation (A) and migration (B) of TP-overexpressing cells.** Cells were incubated for 24 h under hypoxia in the presence of 1 mM Thd. Scratch assay was performed under hypoxic conditions. * p<0.05 NCI-TP vs NCI-EV, # p<0.05 normoxia vs hypoxia.
(PDF)

Figure S7 **Effect of TP overexpression on angiogenic potential of NCI-H292 cells** ***in vitro*** **.** **A**. NCI-H292 cells were stimulated with 1 mM Thd for 48 h in normoxia and conditioned media were applied on HMEC-1 cells seeded on Matrigel. The number of branchpoints formed by HMEC-1 treated with conditioned media from either empty-vector transduced NCI-H292 cells (NCI-EV) or TP-transduced (NCI-TP) has been calculated. **B**. Increased production of IL-8 in TP-overexpressing NCI-H292 cells stimulated with 1 mM Thd for 24 h (n = 4). * p<0.05 NCI-TP vs NCI-EV.
(PDF)

Figure S8 **Effect of TP overexpression on metastasis** ***in vivo*** **.** **A**. Relative Luc activity in inguinal lymph nodes proximal to tumor xenograft measured *ex vivo* upon sacrifice of the animals by bioluminescence imaging on IVIS Lumina II Imaging System (Caliper Life Science) [Centre d'Imagerie du Petit Animal TAAM UPS44, CNRS, Orléans] following intraperitoneal injection of luciferin (150 µL, 5 mg/mL). (n = 7 for NCI-EV, n = 9 for NCI-TP).
(PDF)

Figure S9 **Effect of TP overexpression on gene expression in NCI-H292 tumors** ***in vivo*** **.** mRNA expression of angiogenic factors and TNFα in xenograft tumors (n = 5).
(PDF)

File S1 **Supplementary Methods.** Molecular cloning and vector construction.
(DOC)

File S2 **Supplementary Animation NCI-EV.** Animation representing the results of study of migration of NCI-EV cells by videomicroscopy. Frames are microphotographs recorded at time intervals of 30 min. over 24 hours.
(AVI)

File S3 **Supplementary Animation NCI-TP.** Animation representing the results of study of migration of NCI-TP cells by videomicroscopy. Frames are microphotographs recorded at time intervals of 30 min. over 24 hours.
(AVI)
