# Supplementary material for: Regulation and Novel Action of Thymidine Phosphorylase in Non-Small Cell Lung Cancer: Crosstalk with Nrf2 and HO-1
Source: PLoS One. 2014 May 12;9(5):e97070. doi: 10.1371/journal.pone.0097070 (PMC4018251; doi:10.1371/journal.pone.0097070)
Supplement: Figure S1 — Validation of transgene overexpression in NCI-Nrf2 stably transduced cell line. A. Nrf2 mRNA in NCI-H292-Luc-Nrf2 (NCI-Nrf2) cell line developed as described in Materials and Methods. B. HO-1 mRNA in NCI-Nrf2 cell line. * p<0.05 NCI-Nrf2 vs NCI-EV. (PDF) [file pone.0097070.s001.pdf]

Figure S1

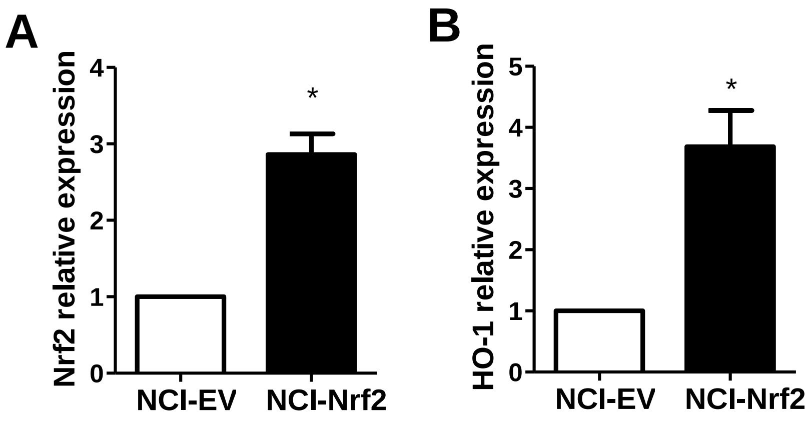

Figure S1. Validation of transgene overexpression in NCI-Nrf2 stably transduced cell line. A. Nrf2 mRNA in NCI-H292-Luc-Nrf2 (NCI-Nrf2) cell line developed as described in Materials and Methods. **B**. HO-1 mRNA in NCI-Nrf2 cell line. * $p<0.05$ NCI-Nrf2 vs NCI-EV
