# Supplementary material for: Regulation and Novel Action of Thymidine Phosphorylase in Non-Small Cell Lung Cancer: Crosstalk with Nrf2 and HO-1
Source: PLoS One. 2014 May 12;9(5):e97070. doi: 10.1371/journal.pone.0097070 (PMC4018251; doi:10.1371/journal.pone.0097070)
Supplement: Figure S2 — Validation of siRNA-mediated knockdown of HO-1 in NCI-H292 cells. NCI-Nrf2 and NCI-EV control cells were transfected with 50 nM siRNA against HO-1 (siHO1) or control scrambled sequence (siSCR) for 72 h leading to downregulation of HO-1 mRNA expression. (n = 4, *p<0.05 NCI-Nrf2 vs NCI-EV, #p<0.05 siHO1 vs siSCR). (PDF) [file pone.0097070.s002.pdf]

Figure S2

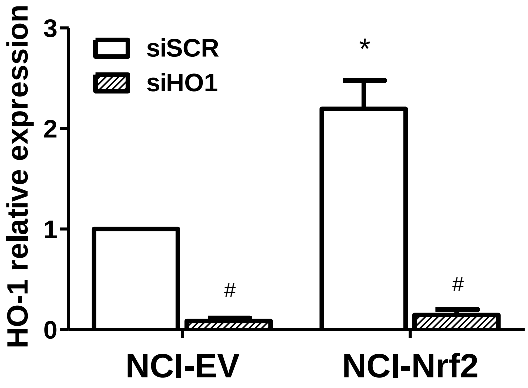

Figure S2. Validation of siRNA-mediated knockdown of HO-1 in NCI-H292 cells. NCI-Nrf2 and NCI-EV control cells were transfected with 50 nM siRNA against HO-1 (siHO1) or control scrambled sequence (siSCR) for 72 h leading to downregulation of HO-1 mRNA expression. (n=4, *p<0.05 NCI-Nrf2 *vs* NCI-EV, #p<0.05 siHO1 *vs* siSCR).
