# Supplementary material for: Regulation and Novel Action of Thymidine Phosphorylase in Non-Small Cell Lung Cancer: Crosstalk with Nrf2 and HO-1
Source: PLoS One. 2014 May 12;9(5):e97070. doi: 10.1371/journal.pone.0097070 (PMC4018251; doi:10.1371/journal.pone.0097070)
Supplement: Figure S3 — Effect of HO-1 products on TP expression. CORM-2 [CORM, CO-releasing molecule - tricarbonyldichlororuthenium(II) dimer], bilirubin and DMSO (used as solvent) were purchased from Sigma Aldrich. Inactive CORM (iCORM) was prepared by overnight evaporation of CORM stock solution. Biliverdin was from MP Bioscience, FeCl3 was from POCh. NCI-EV cells were stimulated with 10 µM CORM, iCORM, biliverdin, bilirubin or FeCl3 for 24 h (n = 3). (PDF) [file pone.0097070.s003.pdf]

Figure S3

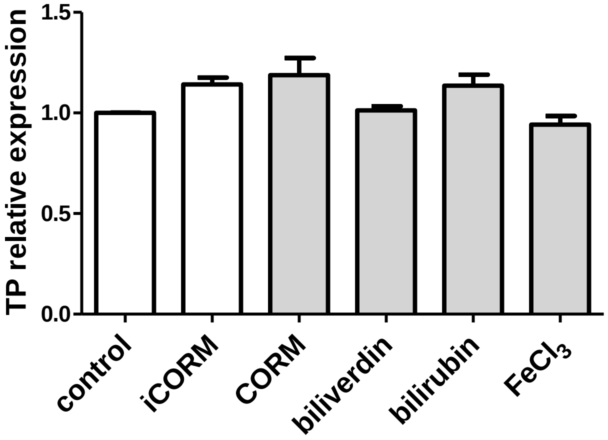

Figure S3. Effect of HO-1 products on TP expression. CORM-2 [CORM, CO-releasing molecule - tricarbonyldichlororuthenium(II) dimer], bilirubin and DMSO (used as solvent) were purchased from Sigma Aldrich. Inactive CORM (iCORM) was prepared by overnight evaporation of CORM stock solution. Biliverdin was from MP Bioscience, $FeCl_3$ was from POCh. NCI-EV cells were stimulated with 10 μM CORM, iCORM, biliverdin, bilirubin or $FeCl_3$ for 24 h (n=3).
