# Supplementary material for: Regulation and Novel Action of Thymidine Phosphorylase in Non-Small Cell Lung Cancer: Crosstalk with Nrf2 and HO-1
Source: PLoS One. 2014 May 12;9(5):e97070. doi: 10.1371/journal.pone.0097070 (PMC4018251; doi:10.1371/journal.pone.0097070)
Supplement: Figure S4 — Effect of TP overexpression on MMP expression. qPCR analysis of MMP-1 and MMP-2 expression in control and TP-overexpressing NCI-H292 cells (n = 4). * p<0.05 NCI-TP vs NCI-EV. (PDF) [file pone.0097070.s004.pdf]

Figure S4

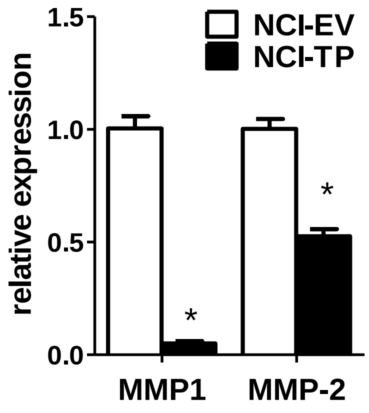

Figure S4. Effect of TP overexpression on MMP expression. qPCR analysis of MMP-1 and MMP-2 expression in control and TP-overexpressing NCI-H292 cells (n=4). * $p<0.05$ NCI-TP vs NCI-EV
