# Supplementary material for: Regulation and Novel Action of Thymidine Phosphorylase in Non-Small Cell Lung Cancer: Crosstalk with Nrf2 and HO-1
Source: PLoS One. 2014 May 12;9(5):e97070. doi: 10.1371/journal.pone.0097070 (PMC4018251; doi:10.1371/journal.pone.0097070)
Supplement: Figure S5 — Effect of TP overexpression on angiogenic potential of NCI-H292 cells in vitro . A. Basal angiogenic potential of TP overexpressing cells. Conditioned media (CM) were collected from unstimulated NCI-H292 cells under normoxia for 24 h. HUVEC spheroids were stimulated with CM or VEGF 10 ng/mL for 72 h (representative experiment, *p<0.05 control vs stimulation). B. Basal mRNA expression of angiogenic factors in TP-overexpressing cells in normoxia. C. mRNA expression of angiogenic factors in NCI-EV cells stimulated with TP products. Cells were stimulated with 200 µM 2-deoxyribose (dR)/2-deoxyribose-1-phosphate (dRP) for 24 h in normoxia. (PDF) [file pone.0097070.s005.pdf]

Figure S5

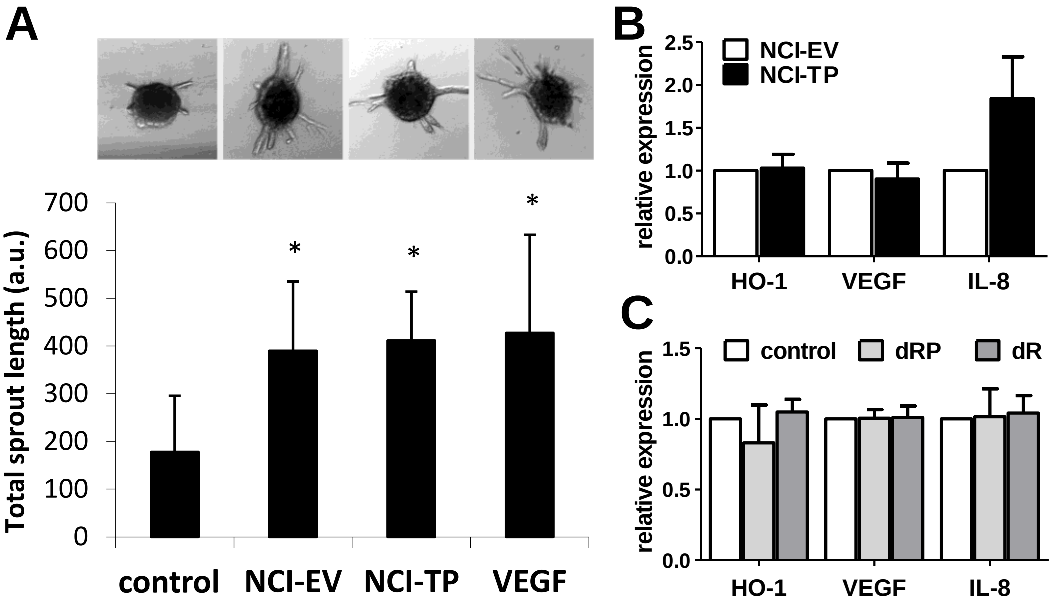

Figure S5. Effect of TP overexpression on angiogenic potential of NCI-H292 cells *in vitro*. A. Basal angiogenic potential of TP overexpressing cells. Conditioned media (CM) were collected from unstimulated NCI-H292 cells under normoxia for 24 h. HUVEC spheroids were stimulated with CM or VEGF 10 ng/mL for 72 h (representative experiment, *p<0.05 control vs stimulation). **B**. Basal mRNA expression of angiogenic factors in TP-overexpressing cells in normoxia. **C**. mRNA expression of angiogenic factors in NCI-EV cells stimulated with TP products. Cells were stimulated with 200 μM 2-deoxyribose (dR) / 2-deoxyribose-1-phosphate (dRP) for 24 h in normoxia.
