# Supplementary material for: Regulation and Novel Action of Thymidine Phosphorylase in Non-Small Cell Lung Cancer: Crosstalk with Nrf2 and HO-1
Source: PLoS One. 2014 May 12;9(5):e97070. doi: 10.1371/journal.pone.0097070 (PMC4018251; doi:10.1371/journal.pone.0097070)
Supplement: Figure S6 — Effects of hypoxia and TP substrate on proliferation (A) and migration (B) of TP-overexpressing cells. Cells were incubated for 24 h under hypoxia in the presence of 1 mM Thd. Scratch assay was performed under hypoxic conditions. * p<0.05 NCI-TP vs NCI-EV, # p<0.05 normoxia vs hypoxia. (PDF) [file pone.0097070.s006.pdf]

Figure S6

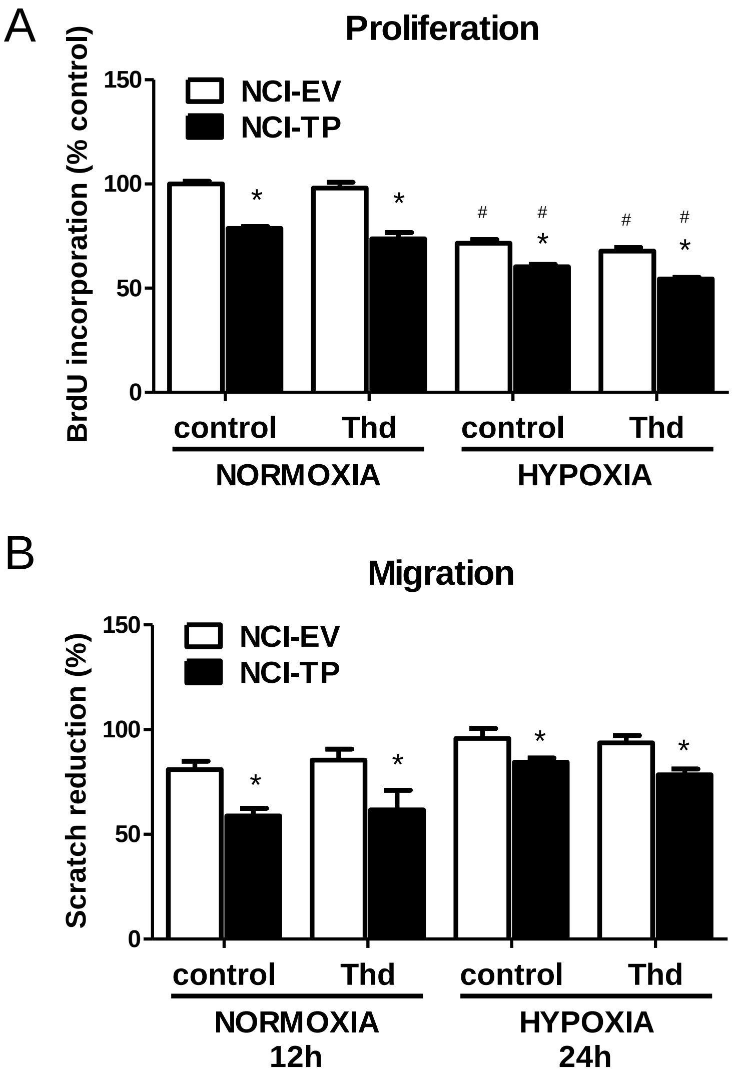

Figure S6. Effects of hypoxia and TP substrate on proliferation (A) and migration (B) of TP-overexpressing cells. Cells were incubated for 24 h under hypoxia in the presence of 1 mM Thd. Scratch assay was performed under hypoxic conditions. * p<0.05 NCI-TP vs NCI-EV, # p<0.05 normoxia vs hypoxia
