# Supplementary material for: Regulation and Novel Action of Thymidine Phosphorylase in Non-Small Cell Lung Cancer: Crosstalk with Nrf2 and HO-1
Source: PLoS One. 2014 May 12;9(5):e97070. doi: 10.1371/journal.pone.0097070 (PMC4018251; doi:10.1371/journal.pone.0097070)
Supplement: Figure S7 — Effect of TP overexpression on angiogenic potential of NCI-H292 cells in vitro . A. NCI-H292 cells were stimulated with 1 mM Thd for 48 h in normoxia and conditioned media were applied on HMEC-1 cells seeded on Matrigel. The number of branchpoints formed by HMEC-1 treated with conditioned media from either empty-vector transduced NCI-H292 cells (NCI-EV) or TP-transduced (NCI-TP) has been calculated. B. Increased production of IL-8 in TP-overexpressing NCI-H292 cells stimulated with 1 mM Thd for 24 h (n = 4). * p<0.05 NCI-TP vs NCI-EV. (PDF) [file pone.0097070.s007.pdf]

Figure S7

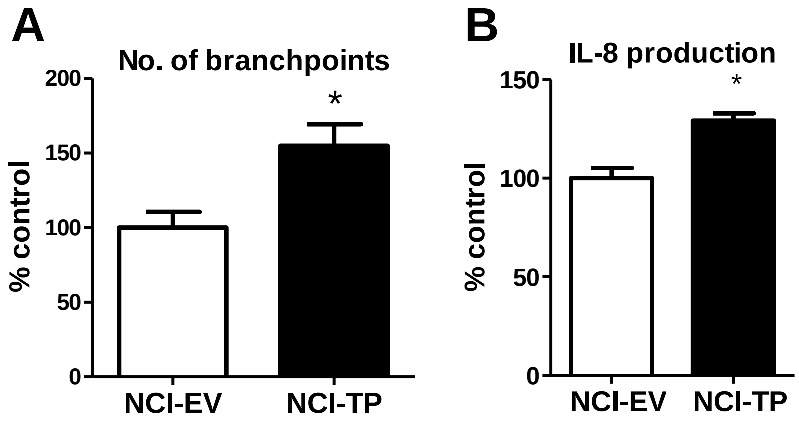

Figure S7. Effect of TP overexpression on angiogenic potential of NCI-H292 cells *in vitro*. **A**. NCI-H292 cells were stimulated with 1 mM Thd for 48 h in normoxia and conditioned media were applied on HMEC-1 cells seeded on Matrigel. The number of branchpoints formed by HMEC-1 treated with conditioned media from either empty-vector transduced NCI-H292 cells (NCI-EV) or TP-transduced (NCI-TP) has been calculated. **B**. Increased production of IL-8 in TP-overexpressing NCI-H292 cells stimulated with 1 mM Thd for 24 h (n=4). * $p<0.05$ NCI-TP vs NCI-EV.
