# Supplementary material for: Regulation and Novel Action of Thymidine Phosphorylase in Non-Small Cell Lung Cancer: Crosstalk with Nrf2 and HO-1
Source: PLoS One. 2014 May 12;9(5):e97070. doi: 10.1371/journal.pone.0097070 (PMC4018251; doi:10.1371/journal.pone.0097070)
Supplement: Figure S8 — Effect of TP overexpression on metastasis in vivo . A. Relative Luc activity in inguinal lymph nodes proximal to tumor xenograft measured ex vivo upon sacrifice of the animals by bioluminescence imaging on IVIS Lumina II Imaging System (Caliper Life Science) [Centre d'Imagerie du Petit Animal TAAM UPS44, CNRS, Orléans] following intraperitoneal injection of luciferin (150 µL, 5 mg/mL). (n = 7 for NCI-EV, n = 9 for NCI-TP). (PDF) [file pone.0097070.s008.pdf]

Figure S8

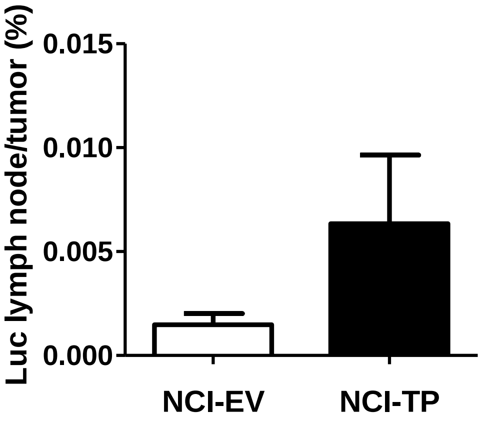

Figure S8. Effect of TP overexpression on metastasis *in vivo*. A. Relative Luc activity in inguinal lymph nodes proximal to tumor xenograft measured *ex vivo* upon sacrifice of the animals by bioluminescence imaging on IVIS Lumina II Imaging System (Caliper Life Science) [Centre d'Imagerie du Petit Animal TAAM UPS44, CNRS, Orléans] following intraperitoneal injection of luciferin (150 µL, 5 mg/mL). (n=7 for NCI-EV, n=9 for NCI-TP).
