# Supplementary material for: Regulation and Novel Action of Thymidine Phosphorylase in Non-Small Cell Lung Cancer: Crosstalk with Nrf2 and HO-1
Source: PLoS One. 2014 May 12;9(5):e97070. doi: 10.1371/journal.pone.0097070 (PMC4018251; doi:10.1371/journal.pone.0097070)
Supplement: Figure S9 — Effect of TP overexpression on gene expression in NCI-H292 tumors in vivo . mRNA expression of angiogenic factors and TNFα in xenograft tumors (n = 5). (PDF) [file pone.0097070.s009.pdf]

Figure S9

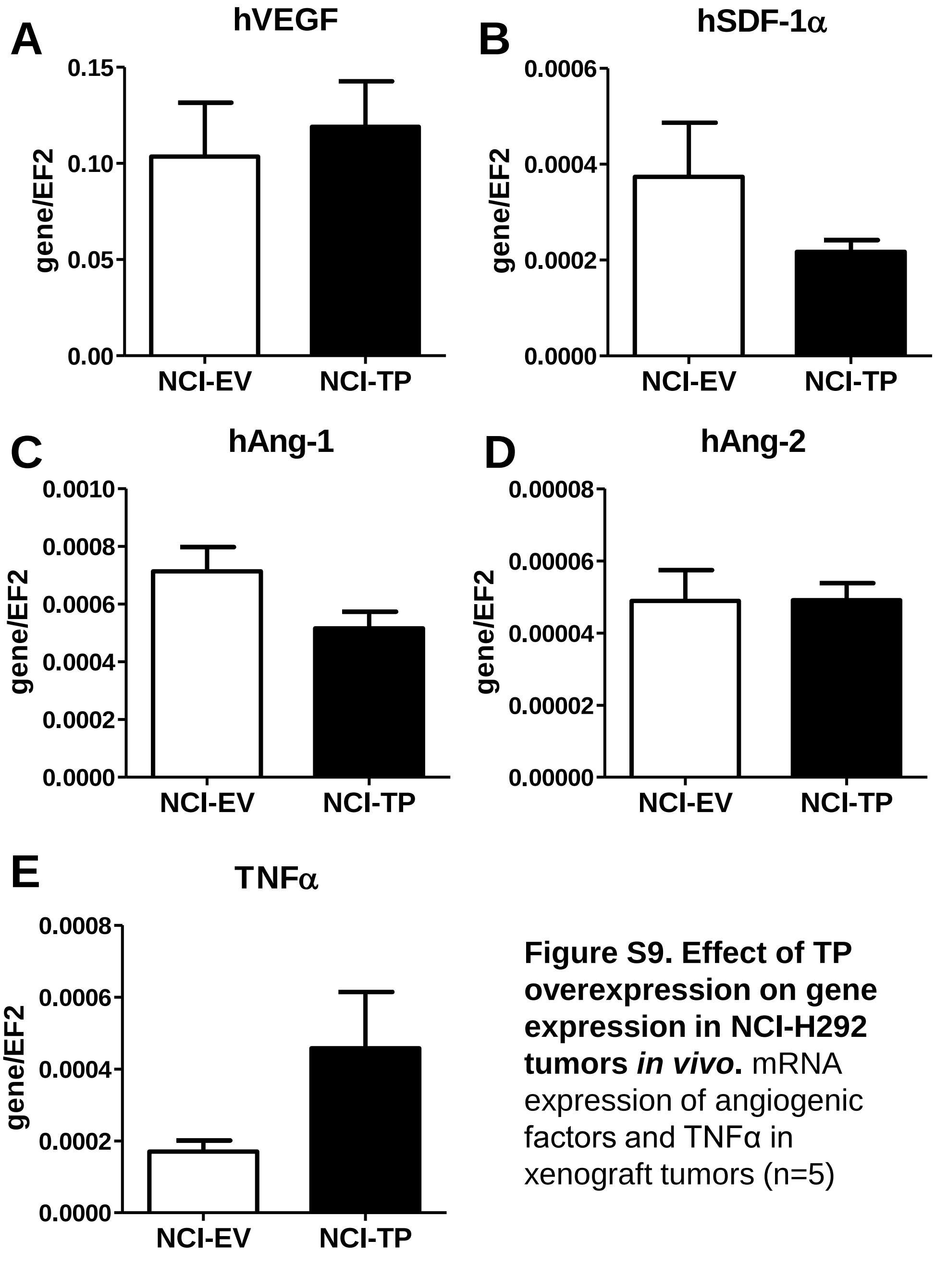

Figure S9. Effect of TP overexpression on gene expression in NCI-H292 tumors *in vivo*. mRNA expression of angiogenic factors and TNFα in xenograft tumors (n=5)
