# Supplementary material for: Regulation and Novel Action of Thymidine Phosphorylase in Non-Small Cell Lung Cancer: Crosstalk with Nrf2 and HO-1
Source: PLoS One. 2014 May 12;9(5):e97070. doi: 10.1371/journal.pone.0097070 (PMC4018251; doi:10.1371/journal.pone.0097070)
Supplement: File S1 — Supplementary Methods. Molecular cloning and vector construction. (DOC) [file pone.0097070.s010.doc]

Supplementary Methods

**Molecular cloning and plasmid amplification**

All restriction enzymes and T4 ligase were from New England BioLabs – reactions were carried out according to manufacturer’s instructions. Plasmids were amplified in MaxEfficiency DH5α *E. coli* strain (Invitrogen) and purified using QiaPrep Midi Kit (Qiagen) according to vendor’s protocol.

**Construction of RV plasmids for overexpression of Nrf2 and TP**

Human Nrf2 cDNA was excised from pEF(Blue)-Nrf2 plasmid (kindly provided by Dr. J.A. Johnson, Division of Pharmaceutical Sciences, University of Wisconsin-Madison, USA) with NotI and ligated to pLNCX2 (Clontech) digested with the same enzyme. Correct orientation of the insert in resulting construct pLNCX2-Nrf2 was verified by restriction analysis.

Human TP cDNA was subcloned from pBK-RSV-TP (kindly gifted by Dr. S. Liekens, Rega Institute, K.U. Leuven, Belgium) into pcDNA3.1 using KpnI and XhoI and next ligated with pLNCX2 after digestion with HindIII. Correct orientation of the insert in resulting construct pLNCX2-TP was verified by restriction analysis.

**Construction of plasmid for production of adenoviral vectors (AdV) for overexpression of TP**

Human TP cDNA was cloned from pBK-RSV-TP into pShuttle-CMV shuttle plasmid from AdEasy System for production of AdV (Stratagene) using KpnI and HindIII restriction sites. Next steps of generation of recombinant AdV harboring TP cDNA were performed according to the AdEasy System protocols.
